# Chronic Obstructive Pulmonary Disease as a Phenotype of Bronchiectasis for Long-Term Clinical Presentation and Treatment

**DOI:** 10.3390/medicina57060579

**Published:** 2021-06-05

**Authors:** Chih-Yi Hsu, Yan-Yuen Poon, Yu-Wei Chen, Meng-Heng Hsieh, Horng-Chyuan Lin, Wen-Feng Fang

**Affiliations:** 1 Department of Internal Medicine, Division of Pulmonary and Critical Care Medicine, Kaohsiung Chang Gung Memorial Hospital, Chang Gung University College of Medicine, Kaohsiung 83301, Taiwan; hsuchiyi@gmail.com; 2 Department of Anesthesiology, Kaohsiung Chang Gung Memorial and Chang Gung University College of Medicine, Kaohsiung 83301, Taiwan; elephant423@gmail.com; 3 Department of Emergency Medicine, Chiayi Chang Gung Memorial Hospital and Chang Gung University College of Medicine, Chiayi 61363, Taiwan; azt911231@gmail.com; 4 Department of Thoracic Medicine, Chang Gung Medical Foundation, Chang Gung University, Taoyuan 33302, Taiwan; mengheng@cgmh.org.tw (M.-H.H.); lin53424@ms13.hinet.net (H.-C.L.); 5 Department of Respiratory Therapy, Kaohsiung Chang Gung Memorial Hospital, Chang Gung University College of Medicine, Kaohsiung 83301, Taiwan; 6 Department of Respiratory Care, Chang Gung University of Science and Technology, Chiayi 61363, Taiwan

**Keywords:** bronchiectasis, chronic obstructive pulmonary disease, COPD-bronchiectasis overlap syndrome, *Pseudomonas*, long-acting β2 sympathomimetic agonists, inhalational corticosteroid

## Abstract

*Background and Objectives:* Bronchiectasis and chronic obstructive pulmonary disease (COPD) often coexist, although the causality is not currently clear. Currently, the clinical influence of COPD on patients with major bronchiectasis over time has not yet been investigated. *Material and Methods:* This retrospective study recruited consecutive patients with bronchiectasis from outpatient clinic between January 2006 and December 2007. Under the setting of quantification with HRCT, patients who should undergo multiple pulmonary function and exercise tests with regularclinic follow-up were included. The final analysis consisted of 66 eligible patients who were evaluated for clinical status, treatment, and sputum culture from up to 10-year electronic medical records. *Results:* Of these 66 patients, 45 (68%) had bronchiectasis without COPD and 21 (32%) had COPD. Patients with COPD group had a higher bronchiectasis extent score (32.21 ± 13.09 points vs. 21.89 ± 10.08 points, *p* = 0.001). Sputum production was reported more frequently by patients with COPD; however, no significant difference was observed after 3 years of follow-up (82.4% vs. 81.6%, *p* = 0.945). Bronchiectasis extent score correlated with positive sputum culture with *Pseudomonas* without a synergistic effect from COPD (odds ratio: 1.06, confidence interval: 1.00–1.12, *p* = 0.031). Regardless of COPD, after 10 years, the proportion of patients using inhaled corticosteroids and/or long-acting β2-agonist between the two groups was not significantly different. *Conclusion:* COPD aggravated bronchiectasis extension, which was correlated with chronic *Pseudomonas aeruginosa* colonisation. Moreover, COPD would affect the medium-term (in 3–5 years) bronchiectasis treatment. Therefore, the COPD phenotype of bronchiectasis could be a clinical predictor of the course of treatment.

## 1. Introduction

A wide range (28–60%) of coexisting bronchiectasis and chronic obstructive pulmonary disease (COPD) has been reported since 2000 [1,2,3]. In recent years, the concept of COPD-bronchiectasis overlap syndrome was first mentioned by Hurst et al. [4]. However, there is currently no guideline to cover clinical practice. Though the classic “vicious cycle” hypothesis was not proven, and the causal association between these two diseases was not clear, COPD coexisting with bronchiectasis was usually considered as a phenotype [5]. Most studies were also based on this setting, and a lack of viewpoint, as bronchiectasis is a major disease. 

On pathology, bronchiectasis is ascribed to irreversibly damaged and dilated bronchi. Bronchial derangement and the co-existence of other respiratory diseases further result in abnormal lung function. The features of bronchiectasis include air flow obstruction, gas trapping and diffusion impairment. Therefore, pulmonary function test (i.e., spirometry), plethysmography and lung diffusion capacity can give clinicians the full picture of this disease instead of image study alone [6]. Clinically, bronchiectasis can cause cough, sputum production, and recurrent respiratory infections [7]. Pathogens conclude *Pseudomonas aeruginosa* and *Haemophilus influenzae,* which represent over 50% [8]. Heterogeneity is also one of its characteristics. All these events finally lead to poor quality of life associated with frequent hospitalizations related to acute exacerbation, or even mortality. 

Moreover, morbidities affect the outcome of bronchiectasis. McDonnell et al. constructed the Bronchiectasis Aetiology Comorbidity Index, which demonstrated the key impact of COPD [9]. The index was used to predict mortality and exacerbation rates in patients with non-cystic fibrosis bronchiectasis. Nevertheless, the influence of COPD the clinical features of patients with bronchiectasis and long-term medications has not yet been evaluated. 

Generally, the severity of bronchiectasis can be assessed using two major tools: bronchiectasis severity index (BSI) and FACED score. Both tools were strong predictors of the 4-year hospitalization rate and mortality [10,11]. Although BSI and FACED scores are useful, they are still not easy to apply to outpatient clinic patients due to the details of pulmonary function tests (PFTs), chronic colonisation, and/or past admission history being needed. These two point assessments are similar, and assessment of radiological extension of this disease is concluded in both scoring tables. 

However, quantitative and qualitative radiological features to assess the severity of bronchiectasis were not new. Several studies have demonstrated four different scoring systems, including the Reiff, Bhalla, Brody, and Robinson scoring systems. Among these four systems, Brody et al. mentioned the most detailed scoring system for evaluating extension [12,13,14].

As the extension of bronchiectasis and COPD were both confirmed as clinical predictors, we investigated the impact of COPD on patients with bronchiectasis for up to 10 years, and the correlation between the extent of bronchiectasis and clinical prognosis.

## 2. Materials and Methods

### 2.1. Setting

The study was approved by the Institutional Review Board of Chang Gung Memorial Hospital (IRB No.202100395B0; approval date: 9 April 2021). The IRB also approved the waiver of the participants’ consent. This study was conducted at the Linkou Chang Gung Memorial Hospital, a 4000-bed tertiary teaching hospital in Northern Taiwan. Patients were recruited from the outpatient clinic of chest medicine.

### 2.2. Study Design

This retrospective study initially included 135 consecutive patients who visited our clinic with a diagnosis of bronchiectasis between January 2006 and December 2007. Bronchiectasis was diagnosed using high-resolution computed tomography (HRCT) where at least one of the following criteria was met: (1) the internal diameter of the bronchus was greater than that of the adjacent pulmonary artery, (2) a lack of tapering of the bronchial lumen toward the periphery, or (3) visualization of the bronchus within 10 mm of the pleural space. 

### 2.3. Inclusion and Exclusion Criteria

**Figure 1:**
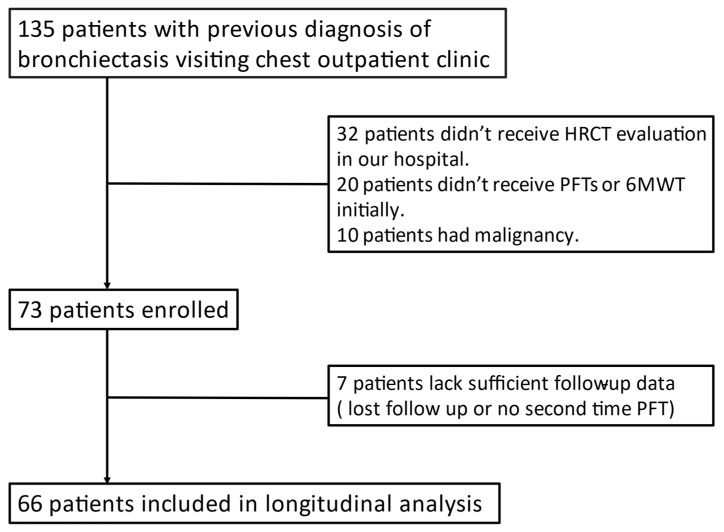
Flowchart.

All patients underwent PFTs and a 6-min walk test (6MWT) initially and at least once repeat for follow-up. Patients without complete examination of baseline data were excluded. If the patients had an underlying malignancy, they were filtered out. Depending on previous ICD code diagnosis and PFT results, the patients could be divided into two different groups. Group 1 comprised patients with bronchiectasis only, and Group 2 consisted of patients with bronchiectasis who met the criteria for chronic obstructive pulmonary disease. The criteria for COPD were established according to the Global Initiative for Chronic Obstructive Lung Disease (GOLD) 2019 guideline, according to clinical history, symptoms, and a post-bronchodilator forced expiratory volume in 1 s (FEV1)/forced vital capacity (FVC) ratio <0.70. All patients were diagnosed by a well-experienced pulmonologist and a radiologist in the medical center (Figure 1). 

### 2.4. 6MWT, PFT, and Sputum Culture 

All patients underwent 6MWT (including PFT) under supervision of well-trained technicians at Linkou pulmonary rehabilitation center. 6MWT was performed in accordance with the standard protocol of the American Thoracic Society [15]. Variables from 6MWT, including pulmonary spirometry, walking distance, and oxygen saturation during the examination were recorded. Sputum was collected at clinic for culture if patients complaint for much sputum production (usually daily sputum ≥10 mL) or not much clinical improvement after antibiotic therapy. Patients were told to spit out the sputum into the sample cup by coughing hard in the separated room.

### 2.5. Data collection: Medical Record, Lung Function Test, 6MWT, and Culture 

Background data were extracted from all participants when they were initially recruited. Medical records included general data (age, sex, and body mass index); smoking habits; history; and ongoing treatment. Lung function test and 6MWT included FVC%, FEV1%, FEV1/FVC, 6-min-walking distance, and oxygen saturation at the beginning and end. Longitudinal data were collected. The tests were not repeated regularly for every patient. However, most patients had several repeat lung functions and sputum investigations during follow-up. The medicine prescriptions were tracked from the clinic records. However, few patients were missing during the preset following period (10 years) for unknown reasons. 

### 2.6. Bronchiectasis Extension Score 

A partial Brody scoring system was used in our study. The scoring system consisted of bronchiectasis, bronchiectasis size, mucous plugging, peribronchial thickening, parenchyma, and hyperinflation scores. We only chose the bronchiectasis score (range, 0–12) as the extension score. There were a total of six lobes (including the lingual lobe); therefore, the full score would be 72.

### 2.7. Statistical Analysis

All statistical analyses were performed using SPSS version 22.0 software (IBM Corp., Armonk, NY, USA). Data were tabulated as mean and standard deviation (SD)for quantitative variables and as absolute numbers. The Kolmogorov–Smirnov test was used to analyze the distribution of variables. In the bivariate analysis, variables with a normal distribution were analyzed using the Student’s t-test for independent variables or the Mann-Whitney U test in other cases. Pearson’s correlation or Spearman’s rank correlation was also used in the bivariate analysis. Statistical significance was set at *p* < 0.05.

Categorical variables are presented as numbers. Comparisons between categorical groups were made using the Chi-square analysis. Multivariate analysis with binary logistic regression was applied to predict outcomes (long-term antibiotic use and Pseudomonas colonisation). Receiver operating characteristic (ROC) curves were used to evaluate the predictive power. The threshold of bronchiectasis extent score was identified by comparing the area under the curve. 

## 3. Results

Of the 66 patients with bronchiectasis, 45 (68%) had bronchiectasis alone, and 21 (32%) had COPD. Table 1 shows the comparison of demographic data, baseline clinical performance, and bronchiectasis extension scores between the two groups. No significant differences were observed in demographic features. However, the group without COPD performed better on pulmonary function and 6MWT, though the walking distance was not significantly different (421.71 ± 102.47 vs. 463.95 ± 80.20, *p* = 0.073). 

In Table 2, the bronchiectasis extension score, rate of decline of pulmonary function, and exercise performance were also compared. Bronchiectasis extension score was significantly greater in the patient group with COPD (32.21 ± 13.09 vs. 21.89 ± 10.08 *p* = 0.001). However, neither decline in pulmonary function (ΔFVC/year, ΔFVC predicted percentage/year, ΔFEV1/year, and ΔFEV1 predicted percentage/year) or variation in 6MWT performance (Δ6MWT-distance/year and Δsaturation-loss/year) demonstrated significant differences. 

The clinical conditions of sputum production reported at OPD are described in Table 3. 

Symptoms of sputum production were initially reported more frequently by patients with COPD (20 out of 21); however, no significant difference was observed after 3 years of OPD follow-up. Positive sputum culture with Pseudomonas was not related to the comorbidity of COPD. Although patients with additional COPD had a higher percentage of positive sputum cultures with bacteria, except for *Pseudomonas*, usage of antibiotics was frequent between the two groups. There was no significant difference in the pattern of antibiotic use between the two groups.

The long-term treatment is also compared in Table 3. Patients receiving ICS and/or long-acting β2-agonist (LABA) in different periods are fully enumerated in this table. A higher percentage of patients with bronchiectasis and COPD had been using a combination of ICS and LABA at the initial time (47.6%), after 3 years (58.8%), and after 5 years (64.7%). Within 10 years, the percentage of patients using inhalation for patients with bronchiectasis alone has been increasing. In the 10th year, no significant differences were observed between the two groups. Some patients also received long-acting muscarinic antagonists (LAMA) as combination with LABA and/or ICS. No patient use LAMA as solitary inhalator treatment.

As shown in Table 4, Pseudomonas was significantly correlated with bronchiectasis extension score (*p* = 0.031). A bronchiectasis extension score of 24.12 may be a cut-off point, which could be a predictive factor for Pseudomonas incidence in patients with bronchiectasis.

The correlation between HRCT score and clinical performance (PFT and 6MWT) is shown in Table S1. There was no significant correlation between HRCT score and clinical prognosis of the pulmonary function test or the 6MWT.

## 4. Discussion

Rosa et al. reported that COPD had an impact on patients with bronchiectasis on disease extension [16]. COPD generally coexists with emphysema. In a previous study, different severities of dilated bronchi and alveolar could be observed through HRCT [12]. Although the causal association was not proven, patients with COPD had a high possibility of developing bronchiectasis [17]. This tendency was verified in this study. Patients with bronchiectasis and COPD primary had a greater extent of bronchiectasis.

Nicotra et al. and King et al. reported that adult nonsmoker patients with non-cystic fibrosis bronchiectasis had an accelerated loss of pulmonary function (FEV1), which was more than expected in the general population [18,19]. Meanwhile, the rate of decline of lung function was greater than age-related change in a substantial proportion of patients with COPD, even after smoking cessation. Surprisingly, COPD had no synergistic effect on patients with bronchiectasis in our study, although COPD is one of the factors that deteriorates lung function in patients with bronchiectasis [20]. However, we only analyzed the progress of spirometry, lacking data of plethysmography and lung diffusion test for making complete evaluation of pulmonary function. 

A retrospective study in 2006 indicated that almost 80% of patients with bronchiectasis were affected by daily sputum production [21]. Bronchiectasis with no sputum, known as “dry bronchiectasis”, do exist; however, it is not common. Generally, chronic sputum production and cough are features of bronchiectasis, similar to COPD. It is not difficult to realize that patients with both diseases had a higher proportion of sputum production at the beginning of recruitment. However, after 3-year observation, the proportion of patients with daily sputum production in the bronchiectasis alone group had increased and showed no significant difference from the group with both diseases. The result implied that COPD had a significant impact on the clinical features of bronchiectasis initially; although sputum production was recorded in most bronchiectasis cases in years. However, in our study, we simply recorded the presence of sputum. Long-term longitudinal studies could be performed with a more detailed quantity and quality of sputum to clarify the impact of COPD. 

Bacterial infection plays an indispensable role in the clinical course of COPD and bronchiectasis. In previous cross-sectional studies, *P. aeruginosa* was isolated from the sputum of 13–20% of adults with COPD [22,23]. The latter have a higher than average risk of developing lung infections. However, these studies did not exclude comorbid bronchiectasis. Although accurate results with the prevalence of bronchiectasis in patients with COPD were not available, conflicting results ranging from 4% to 72% have been analyzed in several studies [17]. In our study, bronchiectasis contributed more to the chronic colonisation rate of PA than COPD (Table 4). That is, COPD had no direct association with Pseudomonas infection. COPD aggravated bronchiectasis extension which correlated with chronic Pseudomonas colonisation. 

In the study by Lynch et al., the bronchiectasis score was correlated with the growth of PA [24]. The original grading system in the study was similar to the Bhalla scoring system. However, the assessment focused more on the severity of the morphology; however, not the extent. Alternatively, other patients with cystic bronchiectasis had a higher risk of growing Pseudomonas from sputum. In this study, we applied a part of the Brody Scoring System, which only considered the extent. Our results revealed that not only did the morphology affect the growth of PA, but the extent of bronchiectasis did. Multiple logistic regression analysis revealed that bronchiectasis extent score was an independently associated factor and was not associated with COPD. Interestingly, we further analyzed the extent score using the ROC curve, and the cut-off point was 24.12. The value was approximately the full score of the two lobes (12 points ×2). The cut point with two lobes was consistent using the BSI and FACED indices. Using these tools, Martinez Garcia MA and Chalmers simply set the same cut point by the number of affected lobes (≥3 or <3) without further considering the morphological findings.

It is reasonable to provide stable patients with oral empiric antibiotics depending upon symptomatic changes caused by either exacerbation of bronchiectasis or COPD. According to the features of chronic bronchial infection related to bronchiectasis, long-term inhaled or oral antibiotic therapy is needed. For bronchiectasis, the European Respiratory Society guidelines released in 2017 suggest a 14-day course of antibiotics based on expert consensus [25]. In contrast, the 2019 GOLD Guidelines for COPD suggested a shorter duration of 5–7 days. In our study, patients with bronchiectasis and COPD had a higher percentage (81%) of long-term antibiotic therapy than those without COPD (64%). However, this difference was not statistically significant. Alternatively, COPD did not affect the pattern of antibiotic use, which depends mainly on bronchiectasis. Conversely, Martinez-Garcia reported that bronchiectasis in COPD has been recognized as a different clinical COPD phenotype with greater symptomatic severity, more frequent chronic bronchial infection and exacerbations, and poor prognosis [17].

ICS plays an important role as a first-line treatment for asthma and reduces exacerbations in COPD [26]. A review indicated that ICS reduced sputum volume in a population with bronchiectasis; however, it did not improve lung function or exacerbation frequency [27]. Furthermore, efficacy and side effect for different ICS was also investigated. A review in 2014 mentioned that combination of fluticasone and salmeterol had more risk of developing pneumonia but less reduction of exacerbation than combination of budesonide and formoterol. [28] ICS or LABA were not the drugs recommended by current guidelines routinely but by the experience of experts. Most patients in our study received medication with a combination of ICS and LABA (budesonide/formoterol or fluticasone/salmeterol). A retrospective study published by Jeong et al. suggested that patients with bronchiectasis can benefit from long-term bronchodilator therapy, regardless of whether the bronchodilator response is positive at baseline [29]. Martínez-García et al. compared medium-dose budesonide (640 μg) plus formoterol with high-dose budesonide (1600 μg) in patients with non-CF bronchiectasis in a 12-month randomized, double-blind, parallel-group trial [30]. They found that patients receiving dual therapy had less dyspnea, rescue β-agonist inhalations, and cough days, although lung function, exacerbation frequency, and chronic bacterial colonisation were not different between the two study groups. Despite the lack of further studies, patients with bronchiectasis appeared to benefit from both drugs. 

Under moderate evidence level, ICS were not suggested to be routinely offered without COPD. Likewise, a long-term bronchodilator was not recommended unless it presented significant breathlessness at the grade D evidence level. The use of bronchodilators or ICS in patients with bronchiectasis and coexisting COPD or asthma should follow the guideline recommendations for COPD or asthma [31]. Instead of judging by patient’s clinical status alone, using plethysmographymight help clinicians to make medicine more precise for bronchiectasis patients. For instance, Sentus et al. pointed out the different impact on inspiratory capacity between long-acting antimuscarinic (tiotropium) and budesonide/formoterol for COPD patients. In the initial recruiting period, patients with bronchiectasis and COPD had more frequent use of LABA or ICS. A significant difference was observed consistently after 5 years. However, according to the medical records after 10 years, the comparison demonstrated no significant difference. In particular, four patients in the bronchiectasis group had developed COPD in the 10th year. The percentage of patients using LABA/ICS increased to 40% in the bronchiectasis alone group. The treatment for bronchiectasis appeared to step closer to COPD over time. 

To date, to the best of our knowledge, no study has investigated the correlation between bronchiectasis extension score and pulmonary function decline. A small sample study in 2002 demonstrated that groups of patients with more extensive bronchiectasis showed a significant decline in FEV1 [32]. Other than this, a controversial result regarding the relationship between pulmonary function decline and chronic PA colonisation was mentioned [5,33]. Although the correlation between bronchiectasis extension score and positive PA colonisation was confirmed in our study, the initial extension score was not related to the progression of pulmonary function. 

There were still some limitations to this study. First, this was a retrospective study with small sample size. It’s difficult to add more subgroups for comparing the trend of specific inhalatory treatment. Second, complete lung function examination including plethysmography and lung diffusion capacity was lack. Questionnaire, dyspnea scales or psychophysical scales such as COPD assessment test (CAT), Modified British Medical Research Council (mMRC) Questionnaire or Borg exertion scale were also important to quantify respiratory symptoms or for calculating BSI and FACED, but it was impossible to re-collect these data. Prospective study with larger sample size which including complete lung function tests and life quality evaluation would help clinicians to clarify the spectrum and interaction between these two diseases. 

## 5. Conclusions

COPD aggravated bronchiectasis extension which correlated with chronic Pseudomonas colonisation. Patients with both diseases had no worse pulmonary functional prognosis than patients with bronchiectasis only. Moreover, COPD only affected the medium-term (3–5 years) bronchiectasis treatment using more appropriate inhalatory treatment. Therefore, the COPD phenotype of bronchiectasis could be a clinical predictor of long-term treatment. The coexistence of these two diseases should not be considered as two independent comorbidities.

## Figures and Tables

**Table 1:** Demographic characteristic and baseline clinical performance between groups with and without chronic obstructive pulmonary disease (COPD).

	Bronchiectasis Patients with COPD (*n* = 21)	Bronchiectasis Patients without COPD (*n* = 45)	*p*-Value
Age	56.95 ± 15.47	58.2 ± 12.81	0.732
Body weight	53.48 ± 9.10	58.29 ± 11.26	0.087
BMI	20.90 ± 2.91	22.62 ± 3.56	0.057
Smoking history	7 (15.6%)	5 (23.8%)	0.499
Past history			
Cardiovascular	4 (8.9%)	0 (0.0%)	0.298
Diabetes mellitus	5 (11.1%)	2 (9.5%)	1.000
TB history (%)	2 (4.4%)	1 (4.8%)	1.000
Lung function test			
FVC	1.63 ± 0.66	2.16 ± 0.79	0.010
FVC%	53.33 ± 17.85	70.58 ± 17.17	0.000
FEV1	0.86 (0.64–1.28)	1.41 (1.17–2.07)	0.000
FEV1%	40.90 ± 18.15	69.04 ± 19.98	0.000
FEV1/FVC	62.70 (51.70–65.65)	78.00 (72.00–83.00)	0.000
6-min walking test			
Walking distance	421.71 ± 102.47	463.95 ± 80.20	0.073
Pre-test saturation	95.00 (92.00–96.00)	96.00 (95.00–97.00)	0.01
Post-test saturation	84.00 (68.75–89.75)	91.00 (85.50–93.00)	0.02
Saturation loss	9.00 (5.50–18.25)	4.50 (3.00–8.00)	0.29

All values as mean±SD; median (IQR) or number and percent. COPD: chronic obstructive pulmonary disease, BMI: body mass index, TB: tuberculosis, FVC: forced vital capacity, FEV1: forced expiratory volume in 1 s.

**Table 2:** HRCT extension score and clinical performance variation.

	Bronchiectasis Patients with COPD (*n* = 21)	Bronchiectasis Patients without COPD (*n* = 45)	*p*-Value
Image study HRCT extension score Clinical performance variation per year	32.21 ± 13.09	21.89 ± 10.08	0.001
Δ FVC/year	−0.62 (−1.80–−0.27)	−0.03 (−0.11–0.00)	0.406
Δ FVC predicted/year	−2.17 (−5.75–−0.86)	−1.00 (−3.98–0.43)	0.444
Δ FEV1/year	−0.03 (−0.08–0.03)	−0.04 (−0.10–0.00)	0.374
Δ FEV1 predicted/year	−1.20 (−5.00–0.86)	−0.83 (−5.12–0.72)	0.940
Δ FEV1/FVC/year	0.41 (−2.70–2.27)	−0.53 (−4.92–−0.08)	0.064
Δ 6MWT-distance/year	−9.92 (−40.04–−5.33)	−1.85 (−20.91–6.82)	0.164
Δ saturation-loss/year	0.33 (−0.54–1.49)	−0.09 (−1.00–1.00)	0.205

All values as mean ± SD; median (IQR) or number and percent. HRCT: High-resolution computed tomography, COPD: chronic obstructive pulmonary disease, BMI: body mass index, TB: tuberculosis, FVC: forced vital capacity, FEV1: forced expiratory volume in 1 s.

**Table 3:** Long term follow-up: sputum production, culture, and inhalation treatment.

	Bronchiectasis Patients with COPD (*n* = 21)	Bronchiectasis Patients without COPD (*n* = 45)	*p*-Value
Sputum production			
Sputum production at recruitment	95.2%	66.7%	0.012
Sputum production after 3 years	82.4%	81.6%	0.945
Sputum culture			
Pseudomonas positive during follow-up	23.8%	24.4%	0.560
Other bacteria positive during follow-up	33.3%	8.9%	0.001
Clinical treatment			
Long term antibiotics use ^1^	80.9%	64.4%	0.174
ICS and/or LABA use after 3 years	47.6%	22.2%	0.020
ICS and/or LABA use after 5 years	64.7%	24.2%	0.012
ICS and/or LABA use after 10 years	57.1%	40.0%	0.195
LAMA use	23.8%	11.1%	0.287

All values as percentages. COPD: chronic obstructive pulmonary disease, ICS: inhaled corticosteroid, LABA: long-acting β2-agonist, LAMA: long-acting muscarinic antagonists. ^1^ antibiotic use over 14 days in continuous 3 months outpatient clinic follow up.

**Table 4:** Binary logistic regression: Pseudomonas positive during follow-up.

	OR	95% CI of OR	*p*-Value
With COPD	1.54	0.44–5.32	0.500
Bronchiectasis extent score	1.06	1.00–1.12	0.031

OR: odds ratio, CI: confidence interval, COPD: chronic obstructive pulmonary disease.

## Data Availability

The data presented in this study are available on request from the corresponding author. The data are not publicly available due to patients’ privacy.

## Supplementary Materials

The following are available online at https://www.mdpi.com/article/10.3390/medicina57060579/s1, Table S1: Correlation between high-resolution computed tomography (HRCT) extension score and clinical performance decline rate.

## References

[1] Du Q., Jin J., Liu X., Sun Y. (2016). Bronchiectasis as a comorbidity of chronic obstructive pulmonary disease: A systematic review and meta-analysis. PLoS ONE.

[2] Martínez-García M.Á., Soler-Cataluña J.J., Sanz Y.D., Serra P.C., Lerma M.A., Vicente J.B., Perpiñá-Tordera M.J.C. (2011). Factors associated with bronchiectasis in patients with COPD. Chest.

[3] Patel I.S., Vlahos I., Wilkinson T.M., Lloyd-Owen S.J., Donaldson G.C., Wilks M., Reznek R.H., Wedzicha J.A. (2004). Bronchiectasis, exacerbation indices, and inflammation in chronic obstructive pulmonary disease. Am. J. Respir. Crit. Care Med..

[4] Hurst J.R., Elborn J.S., De Soyza A. (2015). COPD–bronchiectasis overlap syndrome. Chest.

[5] Blasi F., Chalmers J.D., Aliberti S. (2014). COPD and bronchiectasis: Phenotype, endotype or co-morbidity?. J. Chronic Obstr. Pulm. Dis..

[6] Radovanovic D., Santus P., Blasi F., Sotgiu G., D’Arcangelo F., Simonetta E., Contarini M., Franceschi E., Goeminne P.C., Chalmers J.D. (2018). A comprehensive approach to lung function in bronchiectasis. Respir. Med..

[7] Chung W.-S., Lin C.-L. (2018). Acute respiratory events in patients with bronchiectasis–COPD overlap syndrome: A population-based cohort study. Respir. Med..

[8] King P.T., Holdsworth S.R., Freezer N.J., Villanueva E., Holmes P.W. (2007). Microbiologic follow-up study in adult bronchiectasis. Respir. Med..

[9] McDonnell M.J., Aliberti S., Goeminne P.C., Restrepo M.I., Finch S., Pesci A., Dupont L.J., Fardon T.C., Wilson R., Loebinger M.R. (2016). Comorbidities and the risk of mortality in patients with bronchiectasis: An international multicentre cohort study. Lancet Respir. Med..

[10] Chalmers J.D., Goeminne P., Aliberti S., McDonnell M.J., Lonni S., Davidson J., Poppelwell L., Salih W., Pesci A., Dupont L.J. (2014). The bronchiectasis severity index. An international derivation and validation study. Am. J. Respir. Crit. Care Med..

[11] Martinez-Garcia M., Athanazio R., Girón R., Máiz-Carro L., De la Rosa D., Olveira C., de Gracia J., Vendrell M., Prados-Sánchez C., Gramblicka G. (2017). Predicting high risk of exacerbations in bronchiectasis: The E-FACED score. Int. J. Chronic Obstr. Pulm. Dis..

[12] Brody A.S., Klein J.S., Molina P.L., Quan J., Bean J.A., Wilmott R.W. (2004). High-resolution computed tomography in young patients with cystic fibrosis: Distribution of abnormalities and correlation with pulmonary function tests. J. Pediatr..

[13] Reiff D.B., Wells A.U., Carr D.H., Cole P.J., Hansell D.M. (1995). CT findings in bronchiectasis: Limited value in distinguishing between idiopathic and specific types. Am. J. Roentgenol..

[14] Bhalla M., Turcios N., Aponte V., Jenkins M., Leitman B., McCauley D., Naidich D.J.R. (1991). Cystic fibrosis: Scoring system with thin-section CT. Radiology.

[15] ATS Committee on Proficiency Standards for Clinical Pulmonary Function Laboratories (2002). ATS statement: Guidelines for the six-minute walk test. Am. J. Respir. Crit. Care Med..

[16] De la Rosa D., Martínez-Garcia M.-A., Giron R.M., Vendrell M., Olveira C., Borderias L., Maiz L., Torres A., Martinez-Moragon E., Rajas O.J.P.O. (2017). Clinical impact of chronic obstructive pulmonary disease on non-cystic fibrosis bronchiectasis. A study on 1,790 patients from the Spanish Bronchiectasis Historical Registry. PLoS ONE.

[17] Martinez-Garcia M.A., Miravitlles M. (2017). Bronchiectasis in COPD patients: More than a comorbidity?. Int. J. Chronic Obstr. Pulm. Dis..

[18] Nicotra M.B., Rivera M., Dale A.M., Shepherd R., Carter R.J.C. (1995). Clinical, pathophysiologic, and microbiologic characterization of bronchiectasis in an aging cohort. Chest.

[19] King P.T., Holdsworth S.R., Freezer N.J., Villanueva E., Gallagher M., Holmes P.W. (2005). Outcome in adult bronchiectasis. J. Chronic Obstr. Pulm. Dis..

[20] Ikan A., Adir Y., Stein N., Schneer S., Shteinberg M. (2018). Lung function decline in patients with bronchiectasis. Eur. Respir. J..

[21] King P.T., Holdsworth S.R., Freezer N.J., Villanueva E., Holmes P.W. (2006). Characterisation of the onset and presenting clinical features of adult bronchiectasis. Respir. Med..

[22] Monso E., Garcia-Aymerich J., Soler N., Farrero E., Felez M., Anto J., Torres A. (2003). Bacterial infection in exacerbated COPD with changes in sputum characteristics. Epidemiol. Infect..

[23] Patel I., Seemungal T., Wilks M., Lloyd-Owen S., Donaldson G., Wedzicha J.J.T. (2002). Relationship between bacterial colonisation and the frequency, character, and severity of COPD exacerbations. Thorax.

[24] Lynch D.A., Newell J., Hale V., Dyer D., Corkery K., Fox N.L., Gerend P., Fick R. (1999). Correlation of CT findings with clinical evaluations in 261 patients with symptomatic bronchiectasis. Am. J. Roentgenol..

[25] Polverino E., Goeminne P.C., McDonnell M.J., Aliberti S., Marshall S.E., Loebinger M.R., Murris M., Cantón R., Torres A., Dimakou K. (2017). European Respiratory Society guidelines for the management of adult bronchiectasis. Eur. Respir. J..

[26] King P.J.D. (2007). Is there a role for inhaled corticosteroids and macrolide therapy in bronchiectasis?. Drugs.

[27] McShane P.J., Naureckas E.T., Tino G., Strek M.E. (2013). Non–cystic fibrosis bronchiectasis. Am. J. Respir. Crit. Care Med..

[28] Latorre M., Novelli F., Vagaggini B., Braido F., Papi A., Sanduzzi A., Santus P., Scichilone N., Paggiaro P. (2015). Differences in the efficacy and safety among inhaled corticosteroids (ICS)/long-acting beta2-agonists (LABA) combinations in the treatment of chronic obstructive pulmonary disease (COPD): Role of ICS. Pulm. Pharmacol. Ther..

[29] Jeong H.J., Lee H., Carriere K.C., Kim J.H., Han J.-H., Shin B., Jeong B.-H., Koh W.-J., Kwon O.J., Park H.Y. (2016). Effects of long-term bronchodilators in bronchiectasis patients with airflow limitation based on bronchodilator response at baseline. Int. J. Chronic Obstr. Pulm. Dis..

[30] Martínez-García M.Á., Soler-Cataluña J.J., Catalán-Serra P., Román-Sánchez P., Tordera M.P.J.C. (2012). Clinical efficacy and safety of budesonide-formoterol in non-cystic fibrosis bronchiectasis. Chest.

[31] Hill A.T., Sullivan A.L., Chalmers J.D., De Soyza A., Elborn J.S., Floto R.A., Grillo L., Gruffydd-Jones K., Harvey A., Haworth C.S.J.T. (2019). British Thoracic Society Guideline for bronchiectasis in adults. Thorax.

[32] Sheehan R., Wells A., Copley S., Desai S., Howling S., Cole P., Wilson R., Hansell D.M. (2002). A comparison of serial computed tomography and functional change in bronchiectasis. Eur. Respir. J..

[33] Martínez-García M.A., Soler-Cataluña J.-J., Perpiñá-Tordera M., Román-Sánchez P., Soriano J.J.C. (2007). Factors associated with lung function decline in adult patients with stable non-cystic fibrosis bronchiectasis. Chest.

