# Supplementary material for: Chronic Obstructive Pulmonary Disease as a Phenotype of Bronchiectasis for Long-Term Clinical Presentation and Treatment
Source: Medicina (Kaunas). 2021 Jun 5;57(6):579. doi: 10.3390/medicina57060579 (PMC8226788; doi:10.3390/medicina57060579)
Supplement: Supplementary file 1 [file medicina-57-00579-s001.zip › medicina-1216192-supplementary.pdf]

Supplement

Table.S1 Correlation between high-resolution computed tomography (HRCT) extension score and clinical performance decline rate

PFT and 6MWT	Bronchiectasis patients with COPD (n=21)	Bronchiectasis patients without COPD (n=45)	Overall
△FVC/year	-0.82 (0.75)	0.17 (0.31)	0.05 (0.74)
△FVC predicted/year	-1.99 (0.44)	0.17 (0.31)	0.04 (0.78)
△FEV1/year	-0.35 (0.17)	0.09 (0.60)	-0.56 (0.69)
△FEV1 predicted/year	-0.34 (0.21)	0.15 (0.36)	-0.02 (0.89)
△FEV1/FVC/year	-		
△6MWT-distance/year	0.32 (0.45)	-0.07 (0.98)	-0.17 (0.92)
△saturation-loss/year	0.08 (0.84)	-0.61 (0.77)	0.09 (0.62)

All values are the correlation coefficients (p-values). PFT, pulmonary function test; 6MWT, 6-min walking test; FVC, forced vital capacity; FEV1, forced expiratory volume in 1 s
